# Retraction: Tyrosine Nitration of PA700 Links Proteasome Activation to Endothelial Dysfunction in Mouse Models with Cardiovascular Risk Factors

**DOI:** 10.1371/journal.pone.0286134

**Published:** 2023-05-18

**Authors:** The PLOS ONE Editors

Following the publication of this article [1], concerns were raised regarding results in multiple figures. Specifically,

- The β-actin panels in Figs 3D and 4A appear similar despite representing different conditions.
- The β-actin panels in Figs 3E and 4B appear similar.
- The β-actin panels in Figs 3F and 4C appear similar.

These issues were discussed with the corresponding author and the last author. The corresponding author stated that some of the data underlying the results in this article are no longer available, and they provided files described as the uncropped images underlying some of the panels in the above figures.

The corresponding author acknowledged that the β-actin panel in Fig 4A is incorrect. They provided two different images described as the original blot underlying the correct β-actin panel for Fig 4A, as well as the images described as the underlying blots for β-actin panel in Fig 3D and PA700 panel in Fig 4A. There were discrepancies between the published panels and underlying blots, and the concerns were not fully resolved.

The corresponding author stated that the β-actin panels are identical in Figs 3E and 4B, and Figs 3F and 4C, respectively. They stated that these figures present the same samples, and they apologise that these intentional duplications of the loading controls were not declared in the figure legends. Images described as the underlying blots for PA700 and β-actin in Fig 4C did not appear to match the published panels.

The corresponding author stated that experiments related to Fig 3 and Fig 4 were conducted between the years 2006–2009. They indicated that experiments were performed in batches but did not provide specific information about when each of the presented experiments were performed. Files provided for PA700 and IP: 3-NT/IB:PA700 in Fig 4B suggest that the western blots were performed at different times. The Editors remain concerned regarding the discrepancy in dates and the lack of clarity about whether experimental results presented together were collected at the same time.

In light of the concerns about the reliability of these data, the *PLOS ONE* Editors retract this article.

JX and MHZ did not agree with the retraction. SA responded but expressed neither agreement nor disagreement with the retraction. SW, MZ, and QW either did not respond directly or could not be reached. JX and MHZ stand by the article’s findings.
